# Photoluminescence and Photocurrent from InGaN/GaN Diodes with Quantum Wells of Different Widths and Polarities

**DOI:** 10.3390/nano15020112

**Published:** 2025-01-14

**Authors:** Artem Bercha, Mikołaj Chlipała, Mateusz Hajdel, Grzegorz Muzioł, Marcin Siekacz, Henryk Turski, Witold Trzeciakowski

**Affiliations:** Institute of High Pressure Physics, Polish Academy of Sciences, Sokołowska 29/37, 01-142 Warsaw, Poland; artem@unipress.waw.pl (A.B.); mik@unipress.waw.pl (M.C.); hajdel@unipress.waw.pl (M.H.); gmuziol@unipress.waw.pl (G.M.); msiekacz@unipress.waw.pl (M.S.); henryk@unipress.waw.pl (H.T.)

**Keywords:** nitride LEDs, quantum-confined Stark effect, photoluminescence, photocurrent, screening effects, wide and narrow InGaN quantum wells

## Abstract

We compare the optical properties of four *pin* diode samples differing by built-in field direction and width of the In_0.17_Ga_0.83_N quantum well in the active layer: two diodes with standard *nip* layer sequences and 2.6 and 15 nm well widths and two diodes with inverted *pin* layer ordering (due to the tunnel junction grown before the *pin* structure) also with 2.6 and 15 nm widths. We study photoluminescence and photocurrent in those samples (as a function of excitation power and applied voltage), revealing very different properties due to the interplay of built-in fields and screening by injected carriers. Out of the four types of diodes, the highest photocurrent efficiency was obtained (at reverse voltage) for the wide-well, inverted polarity diode. This diode also showed the highest PL intensity (at positive voltages) of our four samples. Diodes with wide wells have stable emission wavelengths (almost independent of bias and excitation power).

## 1. Introduction

**Figure 1:**
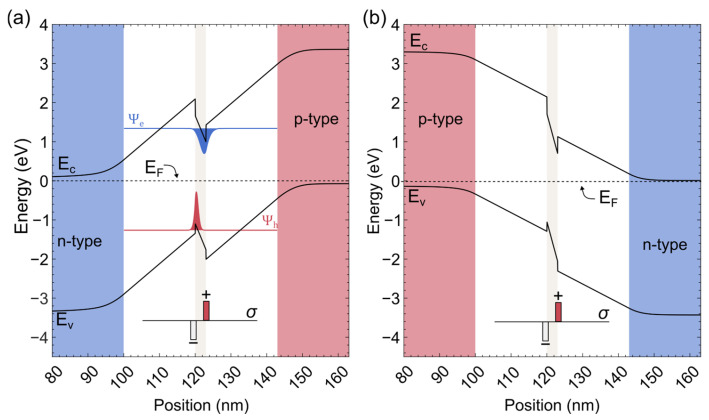
Calculated band structure of a *pin* diode with a narrow quantum well on a Ga-polar substrate, showing (**a**) the standard configuration with the p-type layer on top, (**b**) the inverted configuration with the p-and n-type layers reversed.

Nitride light-emitting diodes (LEDs) and laser diodes (LDs) dominate the UV–blue–green optoelectronics [1,2]. They may also be useful as photodetectors and elements of the multi-junction solar cells [3,4,5]. In a way, photocurrent (PC) and photoluminescence (PL) are competing phenomena [4], since photogenerated carriers can either recombine radiatively or escape from the quantum well by thermionic emission or tunneling (apart from nonradiative recombination, which negatively impacts both PL and PC). InGaN quantum wells are characterized by very strong electric fields (due to spontaneous and piezoelectric polarization), which lead to the quantum-confined Stark effect lowering the interband transition energies and intensities. The presence of strong fields leads to the separation of electrons and holes in the well, which results in the reduction of transition matrix elements, but also in strong screening effects, especially in wide wells. Photoluminescence and photocurrent studies performed as a function of applied voltage (changing the field in the well and barrier) and of excitation power (changing the carrier concentration) reveal a lot of useful information about the electric field and screening effects. This was the main motivation of the present paper: to compare in one study the optical properties of four different LED structures. Since the LEDs grown on Ga-polar substrates are most common, there have been many studies of PL and PC in different structures. For this (standard) polarity, the electric field of the *pn* junction opposes the built-in field in the InGaN/GaN well, to the extent that the resulting field can be completely eliminated [6]. Since N-polar substrates lead to lower efficiency of the structures [7], the practical way to reverse the polarity of the diode is to grow (on a Ga-polar substrate) a thin tunnel junction prior to the diode structure and reverse the order of p- and n-doping [8]. The band structure of LEDs with standard and inverted polarity is shown in Figure 1. Positive (forward) voltage increases the field in the well and decreases the field in the undoped barrier for standard polarity (Figure 1a). For reverse polarity, positive voltage decreases the field both in the well and in the barrier (Figure 1b).

We grew a set of samples with two very different well widths (2.6 and 15 nm, with 17% indium) and two different junction-field directions (*nip* and *pin*). We excited the samples resonantly (i.e., in the well) by a 405 nm laser diode. We also measured the PC spectra using monochromated light from a white-light source. In case of laser excitation, we were able to determine simultaneously the PL and PC. In the present paper, we limit ourselves to CW properties of the diodes, since a comparison of PL and PC for pulsed excitation merits a separate study.

In the following, we first describe the samples and the experimental setup, and we present the results for PL and PC as a function of bias applied to the samples and as a function of excitation power. Finally, we compare the PC spectra for all four samples.

## 2. Materials and Methods

**Figure 2:**
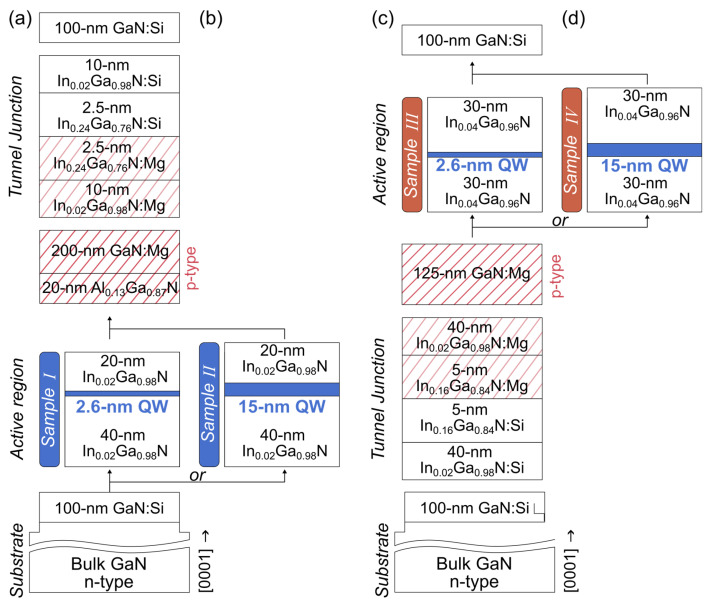
Schematic epitaxial stacks for *nip* and *pin* diode configurations, showing *nip* diodes in (**a**) Sample I with a 2.6 nm QW and (**b**) Sample II with a 15 nm QW and *pin* diodes in (**c**) Sample III with a 2.6 nm QW, and (**d**) Sample IV with a 15 nm QW. Red hatched pattern represents the p-type layers.

The samples were grown by plasma-assisted molecular-beam epitaxy (PAMBE) on bulk Ga-polar c-plane n-GaN substrates. The layers of Sample I were the following: 100 nm n-GaN:Si (Si: 2 × 10^18^ cm^−3^), 40 nm thick In_0.02_Ga_0.98_N lower barrier, 2.6 nm In_0.17_Ga_0.83_N quantum well, 20 nm In_0.02_Ga_0.98_N upper barrier, p-Al_0.13_Ga_0.87_N:Mg electron-blocking layer (EBL), and 200 nm GaN:Mg (Mg: 5 × 10^18^ cm^−3^). Next, an InGaN tunnel junction was grown and capped with a 100 nm n-type GaN:Si on top. The schematic profile of the structure is presented in Figure 2a. The metal contacts formed a rectangular frame on the upper surface so we could illuminate the diode through the opening in metallization. Sample II had an identical structure and composition of the layers but the quantum well was 15 nm wide (Figure 2b). The profile of Sample III is shown in Figure 2c. It contains a tunnel junction prior to the p-type layers so that the *nip* junction for Sample I is replaced by an inverted *pin* junction. Samples III and IV lack the AlGaN electron-blocking layer since it is not necessary in inverted structures [8]. For each sample the quantum well contains In_0.17_Ga_0.83_N, but the barriers contain In_0.02_Ga_0.98_N in Sample I (and Sample II) and In_0.04_Ga_0.96_N in Sample III (and Sample IV). It should also be mentioned that in samples with inverted junctions the quantum well is closer to the sample surface than in samples with standard junctions.

PL was resonantly excited by a 405 nm laser diode using a microscopic objective so that the spot size on the sample was around 50 microns. In each case we label the results by the laser power incident on the sample. PL emission was detected by a SPEX100M (Horiba, Japan) monochromator and a Hamamatsu (Hamamatsu Photonics, Japan) photomultiplier. Photocurrent was measured in the same setup by the voltage drop on a 50-ohm resistor connected in series with the diode (using a Keithley Tektronix, UK voltmeter). For the PC spectra, we used a white-light source (Energetiq LDLS, Hamamatsu Photonics, Japan) passed through a Jobin Yvon microHR (Horiba, Japan) spectrometer. The spot on the sample was also around 50 microns. Light was chopped (at a low frequency of 10 Hz) and the current was detected by a lock-in amplifier. All our measurements were performed at room temperature.

## 3. Results

### 3.1. Results for Samples I and II

**Figure 3:**
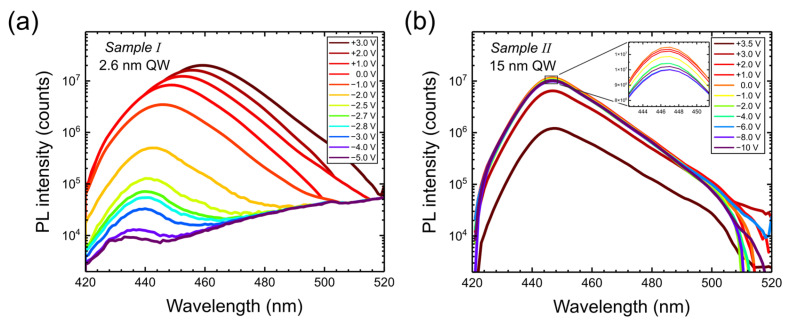
PL spectra versus bias applied to Sample I (**a**) and to Sample II (**b**) for a 2 mW excitation power at 405 nm.

Let us first show the PL spectra at a fixed excitation power as a function of bias in Samples I and II (Figure 3). In the case of a narrow (2.6 nm) well, the emission shifts to longer wavelengths and the intensity increases with increasing bias, while, for the wide well, there is no change of emission wavelength, and the intensity decreases with increasing bias. In Sample I, the PL practically disappears below −3V, while, for Sample II, PL drops above 2V.

**Figure 4:**
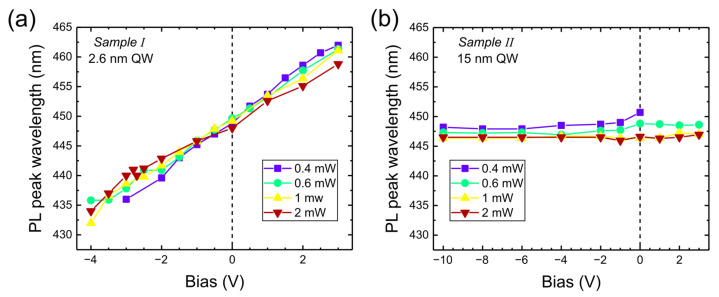
PL emission wavelength versus bias applied to Sample I (**a**) and to Sample II (**b**) for different excitation powers of 405 nm laser.

Similar measurements were taken for excitation powers from 0.4 to 2 mW. The results are summarized in Figure 4. As we can see, the emission wavelength was weakly dependent on excitation power but increased linearly with bias for Sample I and was constant for Sample II.

**Figure 5:**
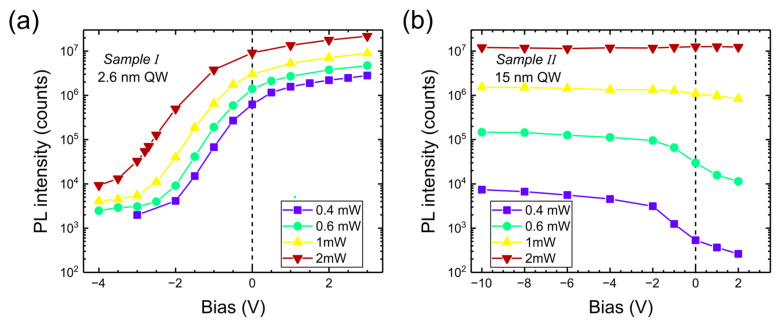
PL intensity (in a logarithmic scale) as a function of bias applied to Sample I (**a**) and to Sample II (**b**) for different excitation powers of 405 nm laser.

These differences between narrow and wide wells (for standard polarization) have been discussed in previous papers [9,10,11], including peculiar properties of wide wells for pulsed excitation. The disappearance of PL signal in narrow wells has been attributed to tunneling escape at reverse bias (the field in the barriers increases, and at high confinement energies in narrow wells, tunneling increases). For positive (forward) bias, the field in the well increases (and the field in the barrier decreases) so that the transition energy is reduced and tunneling is also reduced. The PL in narrow wells is due to transitions between the ground states of electrons and holes. Overlap between their wavefunctions is significant, even at positive bias. This also implies that screening is not very effective in narrow wells because of strong overlap of negative and positive charge. Meanwhile, in wide wells, the electrons and holes in ground states are separated (negligible wavefunction overlap for ground states) so that screening is very effective, but optical recombination is negligible (the charges in the ground states are, therefore, called dark charges). Due to effective screening, the electric field is reduced in the central part of the well, and excited state transitions become dominant. These transitions take place at screened electric fields, so their energy does not change (excited states are also less sensitive to electric fields than ground states). Wide wells are less affected by tunneling, but they are sensitive to bias in terms of PL intensity: for negative bias, the field in the well is reduced and less injected charge goes into ground states (to screen the field) so that the population of excited states increases (for positive bias it is the opposite). In wide wells there are many closely spaced excited states, so the PL emission is a convolution of many transitions. The PL intensities as a function of bias are summarized in Figure 5.

We can see that in the narrow-well sample, the intensity shows a steep increase at negative voltages and slows down at positive voltages. This may be due to the exponential dependence of tunneling rate on barrier thickness. For the wide-well sample, the dependence on excitation power is much stronger than for the narrow well. Apparently, the population of excited states increases relatively faster than the population of ground states.

**Figure 6:**
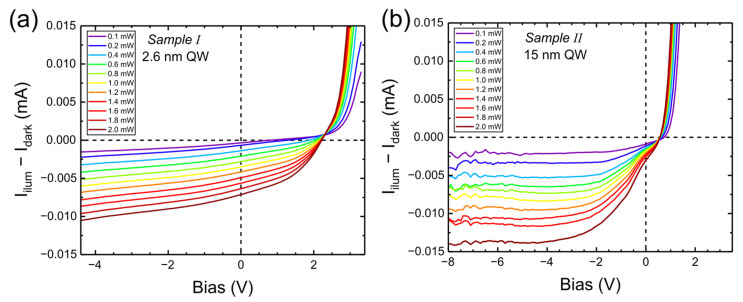
Photocurrent vs. bias for different excitation powers of 405 nm laser for Sample I (**a**) and for Sample II (**b**).

At this point, it is worth looking at the PC from these two samples. We measured the PC under illumination (by the 405 nm laser) and in the dark, as a function of bias, and we subtracted the I–V curves. The results are shown in Figure 6.

The PC is higher in Sample II, which may be due to higher absorption in a wider well. It is interesting that PC changes sign for positive voltages in both samples. The reversal takes place at 2.3 V in Sample I and at 0.8 V in Sample II. We attribute this to the change of band structure by the charge optically pumped into the well. The electrons and holes in the well screen the electric field, which also leads to lower fields in the barrier. This increases the flow of electrons from the n-type layer into the well and the flow of holes from the p-type layer into the well. Therefore, the forward (diffusive) current will be increased by illumination. This effect will be much stronger in a wide well, which is why the reversal takes place at lower voltage in Sample II.

**Figure 7:**
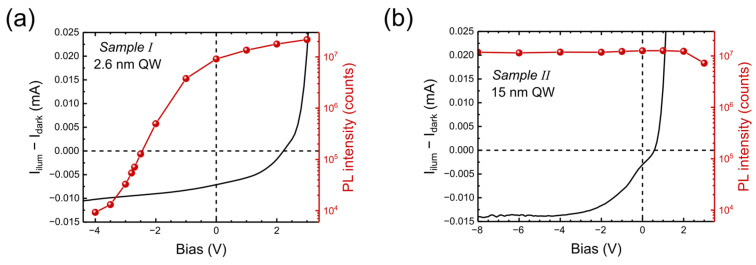
Comparison of PL and PC versus bias for a given excitation power (2 mW) of the 405 nm laser in Sample I (**a**) and Sample II (**b**).

When we compare PC and PL on the same sample, we expect that they compete with each other, i.e., if PL increases, PC should decrease (if we neglect the effect of nonradiative recombination). This is indeed observed for Sample I, with a narrow well (Figure 7a), but it becomes more complicated for Sample II (Figure 7b), where PC is generated by charges in both ground and excited states, while PL originates from excited states only.

Now we turn to a similar investigation of samples III and IV with reverse polarity.

### 3.2. Results for Samples III and IV

In this case, the field in the wells is stronger, since the pn junction field adds to the piezoelectric field in the well (Figure 1b). The field in the barriers should be lower than in samples with standard polarization, since at a given bias the total voltage drop across the diode must be the same.

**Figure 8:**
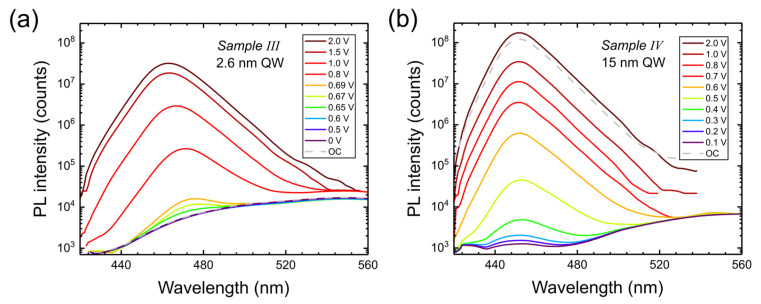
PL spectra at different biases applied to Sample III (**a**) and to Sample IV (**b**) for a 2 mW excitation power at 405 nm. Dashed lines show the PL spectra for open circuit (OC).

**Figure 9:**
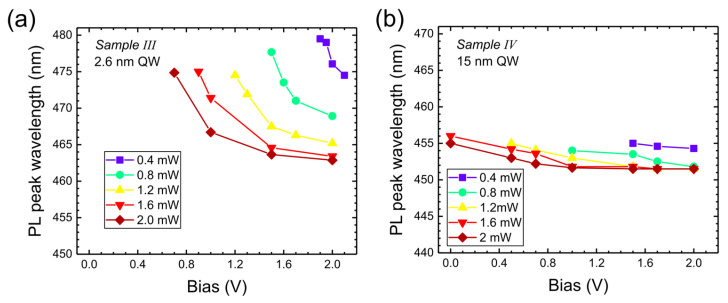
PL emission maxima versus bias applied to Sample III (**a**) and to Sample IV (**b**) for different excitation powers of 405 nm laser.

In Figure 8, we show the spectra as a function of bias for 2 mW excitation by a 405 nm laser (analog of Figure 3). The PL emission wavelengths (at maximum) for various excitation powers are shown in Figure 9 (analog of Figure 4). In this case, applied forward voltage reduces the field in the well, so we observe a blue shift of emission. However, PL appears only above some threshold voltage, increasing for lower excitation powers (Figure 9a). This happens since the reduction of electric field in the well is caused by both screening and positive applied voltage. High electric field eliminates PL emission due to increased tunneling out of the well and lower wavefunction overlap. In the case of a wide well (Sample IV), screening is more effective (since the charges are widely separated) so that PL emission (at 1.6 and 2 mW) already starts at zero voltage. Effective screening also leads to a much smaller wavelength shift with bias in Sample IV (similarly to Sample II).

**Figure 10:**
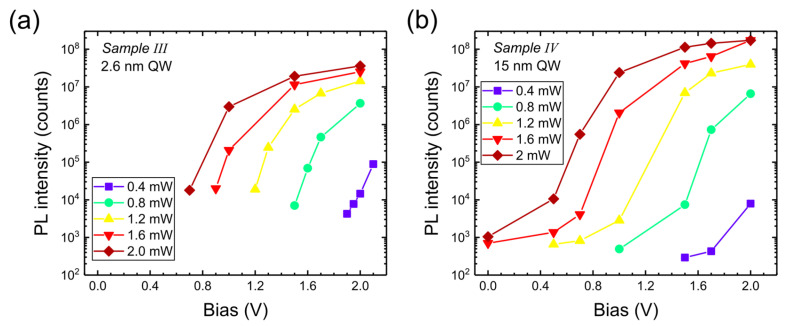
PL intensity as a function of bias applied to Sample III (**a**) and to Sample IV (**b**) for different excitation powers of 405 nm laser.

PL intensities as a function of bias are shown in Figure 10 (analog of Figure 5). The wide-well sample shows the PL signal in a wider range of biases and a steep increase for some range of voltages, decreasing with increased excitation power. Increasing bias leads to increased intensity for both samples because it reduces the field in the well. The highest PL intensities are observed for sample IV (for 2 mW excitation).

**Figure 11:**
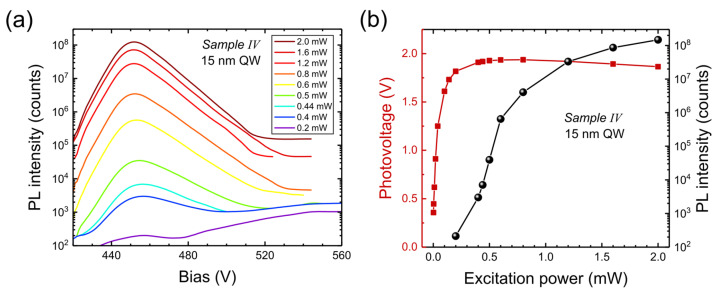
(**a**) PL spectra from Sample IV at open circuit and different excitation powers and (**b**) photovoltage from Sample IV (red symbols) and PL intensity (black symbols) versus excitation power.

At the open circuit there is no PL emission from the quantum well in Sample III, while there is strong emission in Sample IV, similar to the emission at +2 V (Figure 8). Indeed, we found that photovoltage in Sample IV was +2 V (at 2 mW illumination), while, in Sample III, the photovoltage was only +0.44 V, too low to reduce the electric field in the narrow well. This is consistent with the fact that dark charge in the ground state helps to reduce the electric field in a wide well. The PL spectra at the open circuit in Sample IV are shown in Figure 11a, and the photovoltage as a function of excitation power is shown in Figure 11b (together with PL intensity). PL appears above 0.2 mW, while photovoltage is detected at much lower powers.

**Figure 12:**
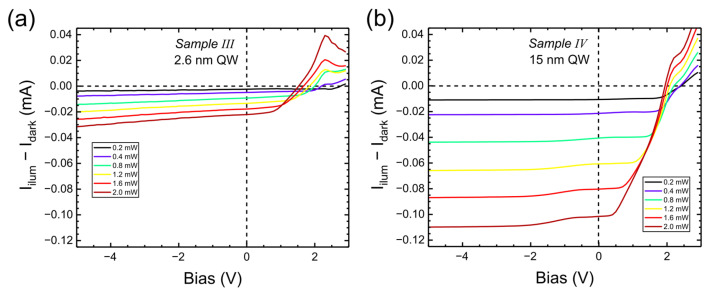
Photocurrent vs. bias for different powers of 405 nm laser for Sample III (**a**) and for Sample IV (**b**).

Now we look at the PC corresponding to the above PL results. Net photocurrent (i.e., I–V under illumination minus I–V in the dark) is shown in Figure 12 for different excitation powers (the analog of Figure 6). For both samples with reverse polarization, the photocurrent changes sign for V > 1.5 V, similarly to Samples I and II.

The PC for reverse voltages is about four times stronger for Sample IV. This implies that such a wide-well diode (with reverse polarity) might be a good candidate for a photodetector in the blue–violet range.

**Figure 13:**
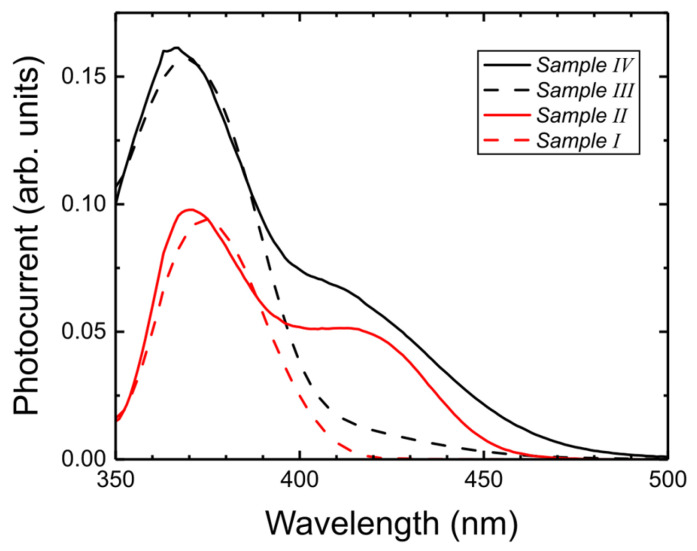
Photocurrent spectra for four samples at zero voltage. Solid lines, wide-well samples (II and IV); dashed lines, narrow-well samples (I and III). Red lines, standard polarization; black lines, reverse polarization.

In order to find the spectral range of photocurrent, we measured the PC spectra in our four samples using monochromatic light and a lock-in amplifier. The frequency of the chopper was set at 10 Hz so that we expect that the results are valid for CW excitation (at high frequencies, the PC spectra for wide wells change significantly). The results for all four samples at zero bias are shown in Figure 13. It has to be mentioned that the photocurrent shown in Figure 6 and Figure 12 for 405 nm illumination was measured at much higher powers than the photocurrent in Figure 13, where the excitation power from monochromatic light was in the μW range. The photocurrent from our samples is not proportional to illumination power (like in Si or GaAs photodiodes) since the photogenerated carriers modify the potential profile by screening the electric field. However, the general features of the spectra should be similar for different light intensities. Please note the much higher PC in the range of quantum-well absorption (400–480 nm) in wide wells. We attribute this to partial screening of the electric field by dark charge, even for low light intensities. In general, wide-well samples (II and IV) show stronger PC and a wider spectral range than narrow-well samples (I and III).

The highest photocurrent (and the widest spectral range) occurs for Sample IV.

## 4. Summary and Conclusions

We have grown LED structures with narrow (2.6 nm) and wide (15 nm) In_0.17_Ga_0.83_N wells in the active layer within the *pin* and *nip* structure (using the tunnel junction before or after the active layer). We compared the PL and PC response of four structures as a function of applied bias and illumination power (by a 405 nm laser). We also measured the PC spectra under low-power monochromatic illumination, and we found superior properties of Sample IV (wide well with inverted structure). Wide wells show specific behavior, which is attributed to the presence of dark charge in the ground states, with a very strong screening effect. Radiative recombination in wide wells occurs between the excited states when the field is screened by dark charge. The emission wavelength in wide wells is fairly stable with excitation power and with bias. Wide wells in inverted structures seem like good candidates for photodetectors and solar cells in the blue–violet region. Recent technological progress in InGaN/GaN photovoltaic devices [12] allows record open-circuit voltage and short-circuit current to be obtained. We expect that using reverse-polarized structures in such devices will lead to further improvements.

## Data Availability

The experimental data shown in the figures is available upon request from Artem Bercha (artem@unipress.waw.pl).
